# A hypocaloric protein-rich diet before metabolic surgery improves liver function in patients with obesity and diabetes: A secondary analysis of a randomized clinical trial

**DOI:** 10.1007/s00423-024-03600-9

**Published:** 2025-01-13

**Authors:** Natalie Krönert, Yusef Moulla, Undine Gabriele Lange, Matthias Blüher, Nicolas Linder, Alexander Fuhrmann, Harald Busse, Anna Linder, Thomas Karlas, Johannes Wiegand, Roland Morgenroth, Lena Seidemann, Arne Dietrich

**Affiliations:** 1 Department of Visceral, Transplant, Thoracic and Vascular Surgery, Leipzig University Hospital, Leipzig, Germany; 2 Department of Endocrinology, Nephrology, Rheumatology, Leipzig University Hospital, Leipzig, Germany; 3 Helmholtz Institute for Metabolic, Obesity and Vascular Research (HI-MAG) of the Helmholtz Zentrum München, University of Leipzig and University Hospital Leipzig, Leipzig, Germany; 4 Department of Diagnostic and Interventional Radiology, Leipzig University Hospital, Leipzig, Germany; 5 Department of Oncology, Gastroenterology, Hepatology, Pneumology and Infectiology, Leipzig University Hospital, Leipzig, Germany; 6 Integrated Research and Treatment Center (IFB) Adiposity Diseases, University Hospital Leipzig, Leipzig, Germany; 7 Clinic for Visceral, Transplant, Thoracic and Vascular Surgery, Leipzig University Hospital, Liebigstr. 20, D-04103 Leipzig, Germany

**Keywords:** Low calory diet, Liver function, Liver volume, Obesity and metabolic surgery

## Abstract

**Purpose:**

Obesity and type 2 diabetes (T2DM) are major risk factors for hepatic steatosis. Diet or bariatric surgery can reduce liver volume, fat content, and inflammation. However, little is known about their effects on liver function, as evaluated here using the LiMAx test.

**Methods:**

In the MetaSurg study (RCT on the effects of different Roux-en-Y gastric bypass (RYGB) limb lengths on diabetes remission in patients with BMI ≥ 27 to ≤ 60 kg/m^2^ and T2DM; trial registration: DRKS00007810, German Clinical Trials Register Freiburg), 24 consecutive patients underwent liver function (LiMAx) and imaging assessments (MRI, transient elastography; TE) before and after diet and surgery. Two weeks before surgery, the patients received a hypocaloric protein-rich diet.

**Results:**

Nine of 18 patients had a pathologic LiMAx value (≤ 315 µg/kg/h) at baseline. After two weeks of diet, LiMAx values improved (*p* = 0.01, paired t test, *n* = 15). LiMAx values further recovered six months after RYGB (*p* = 0.01, paired t test, *n* = 15), which was accompanied by decreased liver volumes (*p* = 0.005, paired t test, *n* = 10), proton density fat fraction (*p* = 0.003, paired t test, *n* = 12), and TE measurements (*p* = 0.032, paired t test, *n* = 14). The need for medical diabetes treatment decreased from 100 to 35%.

**Conclusion:**

Liver function improved after a two-week hypocaloric protein-rich diet and metabolic surgery in patients with obesity and T2DM. These data suggest that a two-week diet for this group of patients prior to abdominal surgery could improve a presumably impaired liver function.

**Supplementary Information:**

The online version contains supplementary material available at 10.1007/s00423-024-03600-9.

## Introduction

Obesity and insulin resistance are main risk factors for metabolic dysfunction-associated steatotic liver disease (MASLD) [1–3]. The global prevalence of MASLD is 30% and is even higher in the presence of obesity [4, 5]. Thus, the majority of patients who undergo bariatric/metabolic surgery are likely affected.

Liver biopsy is the gold standard for MASLD diagnosis and staging. But due to its invasiveness and possible complications, it is unsuited for widespread use as a screening or monitoring tool [6]. Several non-invasive diagnostic tools based on serum parameters or imaging techniques, like magnetic resonance-based proton density fat fraction (PDFF) or ultrasound-based liver elastography combined with attenuation-based steatosis measurement, serve as surrogate techniques [7, 8]. However, the diagnostic accuracy (and for imaging studies also feasibility) of these tests is limited in severely obese patients such as bariatric surgery candidates [9, 10]. Furthermore, laboratory and imaging analyses provide, if at all, only indirect information on the hepatic function that can be impaired even at early stages of MASLD. The LiMAx (maximum liver function capacity) test was developed to assess the liver function capacity non-invasively before major hepatic resections [11] and has been shown to detect liver function impairment more reliably than indocyanine green plasma disappearance rate [12, 13]. It was also validated in bariatric surgery candidates in whom it detected reduced liver function capacities especially in patients with obesity and type 2 diabetes mellitus (T2DM) and proved useful in screening for MASH [14].

At early MASLD stages, hepatic alterations are reversible and can be improved by substantial weight loss following lifestyle interventions or bariatric surgery [15, 16]. Preoperative hypocaloric diets before bariatric surgery can reduce liver volume, hepatic fat content and parameters of inflammation [17]. To the best of our knowledge, there are no data addressing the effect of such a diet and consecutive gastrointestinal bypass surgery on the liver function of patients treated in metabolic surgery programs.

The LiMAx study is a substudy of the prospective randomized clinical trial MetaSurg (Metabolic Surgery for Type 2 Diabetes within BMI range of 27 to 60 kg/m^2^). It analyzed the effects of a preoperative two-week hypocaloric protein-rich diet and consecutive metabolic surgery on liver function and imaging-based MASLD parameters.

## Materials and methods

### Trial design

The LiMAx study was a concomitant scientific project to the MetaSurg study, an open-label prospective randomized parallel group trial that investigated effects of two Roux-en-Y gastric bypass (RYGB) variants with different limb lengths (standard RYGB (stRYGB) and changed limb length RYGB (cllRYGB)) in patients with T2DM in the context of metabolic surgery. The single center trial was conducted at University Hospital Leipzig, Germany and the MetaSurg trial protocol is available in the Supplementary Information (Online Resource 1). Detailed inclusion and exclusion criteria are given in Online Resource 2. After screening, MetaSurg patients were randomized to one of the two surgical arms. The company Humedics GmbH (Humedics GmbH, Marie-Elisabeth-Lüders-Straße 1, 10625 Berlin) granted financial support by covering the test expenses for 30 patients at maximum. Thus, a sample size estimation for power calculations was obsolete. From 21/03/2019 to 29/10/2020, 42 patients were recruited for the two surgically treated study arms of the MetaSurg trial. Twentyfive out of these 42 gave their consent to additionally participate in the LiMAx study. At the beginning of the study, the MetaSurg trial protocol also included an observational control (medical treatment instead of surgery). But due to patient refusal to further participate in the trial after being randomized to the control, this had to be abandoned. Thus, there is no non-surgical control and also no control group without diet, since the LiMAx study was embedded in MetaSurg which required a preoperative diet for all surgical patients. The MetaSurg study is registered at “Deutsches Register Klinischer Studien Freiburg” (German Clinical Trials Register Freiburg; DRKS-ID: DRKS00007810) and was approved by the ethics committee of Leipzig University (153/15-ek). All participants provided written informed consent.

### Interventions

Prior to surgery, all patients were recommended a hypocaloric protein-rich diet for 14 days similar to a previously published protocol [17]. The detailed diet instructions handed out to the participants are given in the Supplementary Information (Online Resource 3). In brief, participants were instructed to consume four protein shakes daily (prepared with protein powder in skimmed milk, buttermilk or water) to achieve a daily protein intake of ~ 70 g and a daily energy intake of ~ 900 kcal. Additional consumption of vegetables was allowed with optional limited supplementation of low-fat milk products or milk alternatives (e.g. soy milk). Diet adherence was monitored by food diaries and estimated by a professional dietician as published before [17]. RYGB was performed as laparoscopic procedure. The size of the stomach was first reduced to a small pouch with a volume of 10–20 cm^3^. Then, the pouch was attached to the small intestine (alimentary limb; AL), bypassing most of the rest of the stomach and the upper part of the small intestine (biliopancreatic limb; BPL). Assigned limb lengths in the stRYGB group were: 150 cm AL / 50 cm BPL for BMI ≤ 50 kg/m² and 170 cm AL / 80 cm BPL for BMI ≥ 50 kg/m². Limb lengths in the cllRYGB group were: 50 cm AL / 150 cm BPL for BMI ≤ 50 kg/m² and 80 cm AL / 170 cm BPL for BMI ≥ 50 kg/m².

### Outcomes

The primary outcome measure was the change in liver function assessed by the LiMAx test after above mentioned diet and surgical procedure. A further aim of the study was to analyze if morphological parameters (liver volume and degree of steatosis assessed by magnetic resonance imaging (MRI) and transient elastography (TE)) reflect the expected functional hepatic changes.

Within six weeks after study enrolment and baseline visit (V1), participants started the diet, and after two weeks, visit 2 (V2) was scheduled on the day before surgery. Postoperative follow-ups took place three (V3) and six (V4) months after surgery. Anthropometric measurements and LiMAx tests were performed at all study visits, MRIs and TE on V1 and V4.

### LiMAx test

The LiMAx test assesses the liver function capacity by metabolization of the ^13^C-labelled test substance methacetin (Methacetin^®^, Humedics GmbH, Berlin, Germany) by the cytochrome P450 (CYP) 1A2 enzyme that is exclusively expressed in the liver [11]. After intravenous bolus injection of 2 mg/kg ^13^C-methacetin, it is metabolized to acetaminophen and the non-radioactive ^13^CO_2_ isotope, which is exhaled. The exhaled air is directed (via mask and tube) into the FLIP device (Humedics GmbH, Berlin, Germany) that detects the ^13^CO_2_/^12^CO_2_ ratio over a period of 60 min and calculates the LiMAx value as maximum delta over baseline. A LiMAx value of > 315 µg/kg/h is considered a normal liver function capacity [18]. The LiMAx maximum liver capacity test is certified for diagnostic use in the European Union and the United Kingdom.

### Transient elastography (FibroScan)

Transient elastography (TE) for the assessment of hepatic steatosis (Controlled Attenuation Parameter; CAP; in dB/m) and fibrosis (Liver Stiffness Measurement; LSM; in kPa) was performed with the FibroScan device (Echosens, Paris, France) according to the manufacturer’s instructions and the European Federation of Societies for Ultrasound in Medicine and Biology (EFSUMB) Guidelines and Recommendations on the Clinical Use of Liver Ultrasound Elastography [19].

### MRI assessment of liver volume and hepatic fat fraction

Abdominal MRIs were performed on a 3 Tesla scanner (Achieva XR, Philips Healthcare, Best, The Netherlands) with a bore diameter of 60 cm using the integrated body coil for signal reception. The protocol included an axial in-phase/opposed-phase gradient echo (Dixon) sequence with images (slice thickness 10 mm) acquired in breath-hold technique (expiration). The liver volume was determined by an in-house segmentation tool (developed under Matlab, The MathWorks Inc., Natick, MA, USA) that was used previously by Karlas et al. [20]. The hepatic fat fraction was estimated by computing the MRI-based proton density fat fraction (PDFF) from the respective Dixon images and averaging the values over three regions of interest placed in liver segments II, IV, and VII.

### Statistical methods

Statistical analysis was performed with Microsoft Excel 2016 and IBM SPSS Statistics version 29. Graphs were created in GraphPad Prism 7.05. For longitudinal data, univariate and multivariate analyses were done with a linear mixed model. Two-sided t test was applied to compare means of paired samples. Associations between continuous variables were tested by Pearson’s correlation. Paired nominal data were analyzed by McNemar test. A *p* < 0.05 was considered statistically significant.

## Results

**Fig. 1:**
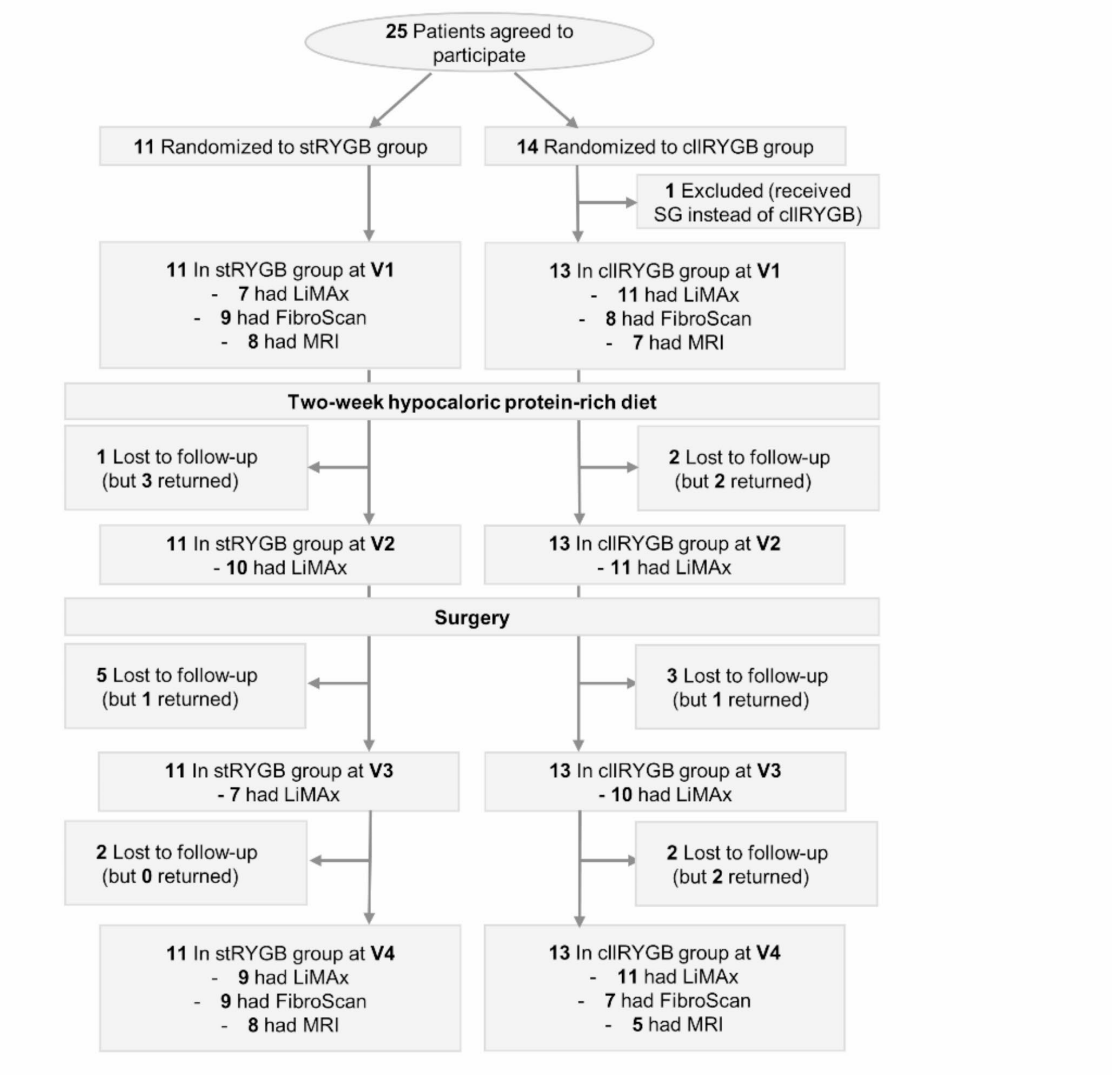
Participant Flow in the LiMAx Study. Abbreviations: stRYGB, standard Roux-en Y gastric bypass; cllRYGB, changed limb length Roux-en Y gastric bypass; SG, sleeve gastrectomy; LiMAx, maximum liver function capacity; MRI, magnetic resonance imaging; V1, baseline visit at participant enrolment in the study; V2, after two-week hypocaloric protein-rich diet on the day before surgery; V3, three months post-surgery; V4, six months post-surgery

From 21/03/2019 to 29/10/2020, 25 out of 42 consecutive patients, who were randomized into one of the two surgical arms of the MetaSurg study, were willing to participate in the LiMAx substudy. The final date for follow-up data collection was 07/07/2021. Data of patients who had at least one preoperative LiMAx test (V1 or V2) were included in the final analyses. One patient had to be excluded after surgery, because a sleeve gastrectomy had to be performed for anatomical reasons instead of the planned cllRYGB, leading to 24 participants being analyzed. The numbers of patients evaluated by LiMAx test on the single study visits were: V1: *n* = 18, V2: *n* = 21, V3: *n* = 17, V4: *n* = 20. Due to restricted hospital access during the COVID-19 pandemic and a temporary bottleneck of methacetin supply, the study experienced markedly varying participant numbers at the different study visits. The study flow chart is shown in Fig. 1. Mean age was 53 (SEM 2.23) years and mean BMI 45.65 (SEM 1.07) kg/m^2^. Sixteen participants were women, 8 were men. All had T2DM. Before start of the diet, mean weight was 132.13 (SEM 3.4) kg and after diet 128.35 (SEM 3.35) kg. Mean weight difference before and after diet was 4.8 (SEM 0.61) kg. Thus, a good overall adherence to the preoperative diet could be concluded. Detailed patient characteristics are given in Table 1. There was no morbidity and no mortality.

**Table 1:** Patient characteristics

	V1	V2	V3	V4
Age (years)	52.63 (2.23) *n* = 24			
Sex				
Men	8 (33%)			
Women	16 (67%)			
BMI (kg/m^2^)	45.65 (1.07) *n* = 24	44.32 (1.04) *n* = 24	36.07 (1.1) *n* = 17	34.09 (0.9) *n* = 23
T2DM				
Insulin treatment	4			3
Insulin treatment + oral medication	8			5
Oral medication	12			0
Dietary treatment	0			16
LiMAx (µg/kg/h)	327.33 (23.3) *n* = 18	349.95 (17.56) *n* = 21	366.18 (22.45) *n* = 17	400.6 (23.33) *n* = 20
PDFF (%)	11.3 (2.56) *n* = 12			2.99(0.53) *n* = 12
Liver volume (ml)	2379 (266.06) *n* = 10			1820.2 (172.81) *n* = 10
CAP (dB/m)	298.36 (15.87) *n* = 14			235.57 (19.39) *n* = 14
LSM (kPa)	11.94 (2.47) *n* = 14			7.39 (1.59) *n* = 14
Weight (kg)	132.13 (3.4) *n* = 24	128.35 (3.35) *n* = 24	104.67 (3.6) *n* = 17	99.26 (3.25) *n* = 23

Abbreviations: V1, baseline visit at participant enrolment in the study; V2, after two-week hypocaloric protein-rich diet on the day before surgery; V3, three months post-surgery; V4, six months post-surgery; BMI, body mass index; LiMAx, liver maximum capacity; PDFF, proton density fat fraction; CAP, continuous attenuation parameter; LSM, liver stiffness measurement

Values are shown as total numbers or mean and standard error of the mean (SEM)

### Impaired liver function recovers after diet and additionally after metabolic surgery

Overall, it could be observed that the mean LiMAx values increased over time (Table 1). At baseline (V1; *n* = 18), 50% of the available LiMAx tests yielded pathological results below the normal range of > 315 µg/kg/h. The rate of pathological liver function capacities was only 15% at six months postoperatively. Of the 18 patients who provided data at V1, 15 also provided data at V4 and only 2 of the original 9 with impaired liver function still had pathological results (*p* = 0.016; McNemar test). For longitudinal analysis of all available LiMAx values, a linear mixed model with LiMAx as dependent variable and time as fixed factor was applied (Table 2). Pairwise comparisons with Bonferroni correction to adjust for multiple testing revealed that LiMAx values 6 months after operation (V4) were significantly higher than prior protein diet (V1) (mean difference − 80.473 µg/kg/h, SEM 19.472, 95% confidence interval (CI) [-133.865, -27.081], *p* < 0.001). Also, Limax values improved significantly 6 months post-RYGB (V4) in comparison to liver function values on the day before surgery (V2) (mean difference − 63.141 µg/kg/h, SEM 18.451, 95% CI [-113.765, -12.517], *p* = 0.007). In this model, there were no differences between pre- (V1) and post-diet (V2) values or 3 months after surgery (V3).

**Table 2:** Longitudinal analysis of all acquired LiMAx values during the study

				95% confidence interval for the difference between means	
	**Mean difference** **(µg/kg/h)**	**SEM**	***p*** **value**	**Lower limit**	**Upper limit**
V1 vs. V2	-17.332	19.327	1.000	-70.315	35.652
V1 vs. V3	-33.186	20.823	0.702	-90.261	23.890
V1 vs. V4	-80.473	19.472	< 0.001*	-133.865	-27.081
V2 vs. V3	-15.854	19.971	1.000	-70.608	38.900
V2 vs. V4	-63.141	18.451	0.007*	-113.765	-12.517
V3 vs. V4	-47.287	20.331	0.143	-103.008	8.434

A linear mixed model analysis with LiMAx as dependent variable and time as fixed factor was applied. Pairwise comparisons between time points were done with Bonferroni correction for multiple testing. *p < 0.05 was considered statistically significant

Abbreviations: V1, baseline visit at participant enrolment in the study; V2, after two-week hypocaloric protein-rich diet on the day before surgery; V3, three months post-surgery; V4, six months post-surgery; SEM, standard error of the mean

To identify factors that influenced the change in liver function, a multivariate analysis was performed by introducing age, sex, type of surgical procedure and weight as covariates with fixed effects into the linear mixed model (Table 3). Thereby only weight had a significant effect on the change in LiMAx values.

**Table 3:** Multivariate analysis of factors influencing LiMAx improvement

				95% confidence interval	
	**Estimate**	**SEM**	***p*** **value**	**Lower limit**	**Upper limit**
Intercept	703.818	162.687	< 0.001*	372.371	1035.265
Age	-1.597	1.499	0.297	-4.691	1.496
Sex	10.370	34.647	0.767	-61.287	82.027
Procedure	46.274	31.215	0.152	-18.434	110.982
Weight	-2.447	1.071	0.028*	-4.620	-0.274

Age, sex, surgical procedure and weight were introduced as covariates with fixed effects into the linear mixed model analysis of all acquired LiMAx values during the study. *p < 0.05 was considered statistically significant

Abbreviations: SEM, standard error of the mean

**Fig. 2:**
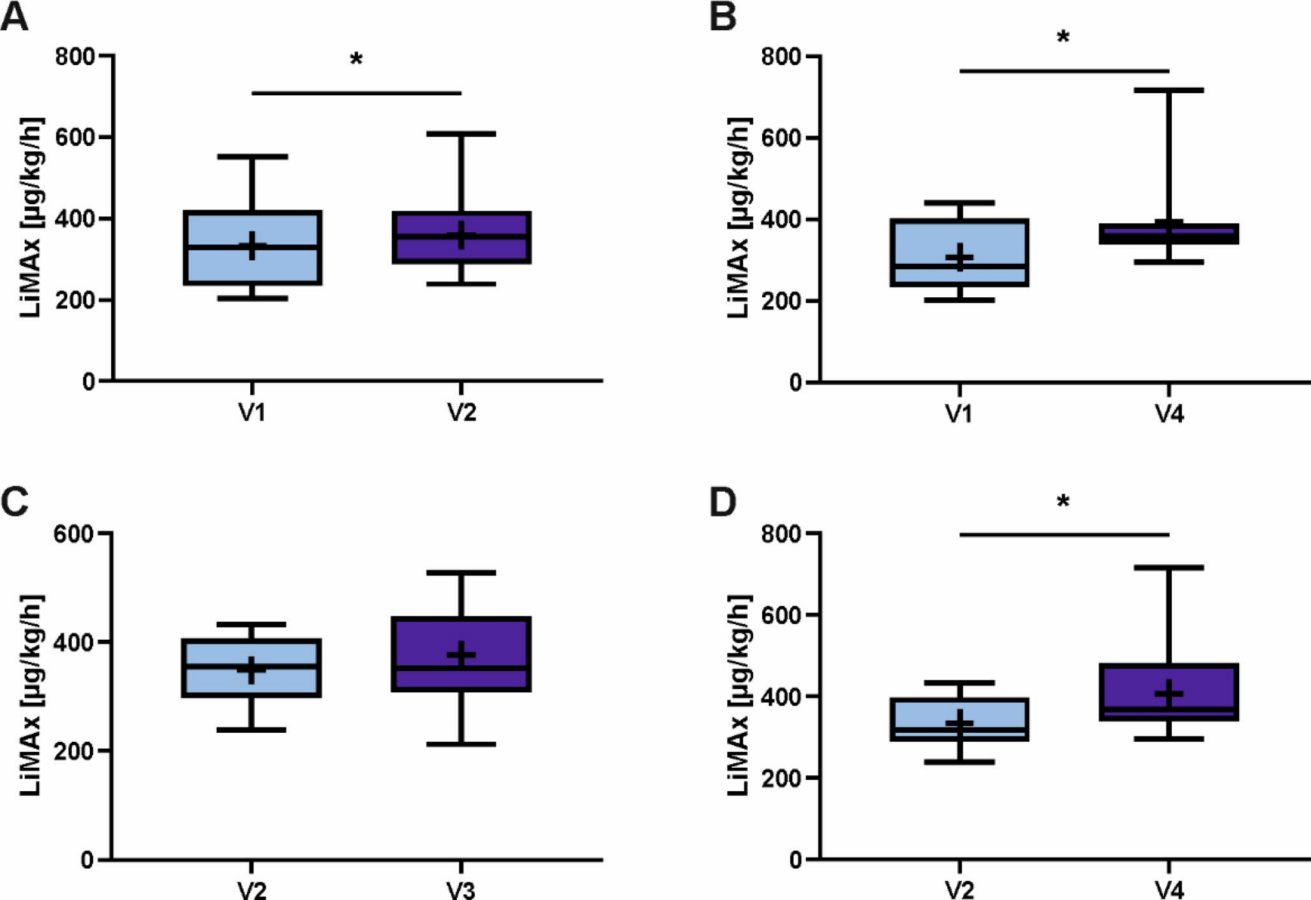
Effects of Diet and Metabolic Surgery on the Liver Function. Maximum liver function capacity (LiMAx) was measured before and after a two-week hypocaloric protein-rich diet (A; *n* = 15, *p* = 0.01), before diet and 6 months after Roux-en-Y gastric bypass (RYGB) surgery (B; *n* = 15; *p* = 0.01), after diet and 3 months after RYGB (C; *n* = 15; *p* = 0.107), after diet and 6 months after RYGB (D; *n* = 18; *p* = 0.006). Data are expressed as boxplots with median as middle line and interquartile range (IQR) as ends of the box, minimum and maximum values as whiskers and a cross marking the mean value. Paired t test, *p* < 0.05 (*). Abbreviations: V1, baseline visit at participant enrolment in the study; V2, after two-week hypocaloric protein-rich diet on the day before surgery; V3, three months post-surgery; V4, six months post-surgery; LiMAx, maximum liver function capacity

Since the number of patients that provided LiMAx data fluctuated greatly between study visits, also direct comparisons were performed with the available cross-sectional sample data using two-sided paired t tests (Fig. 2). Analogously to the results of the longitudinal analysis, LiMAx values improved significantly between V1 and V4 (mean difference − 87.067 µg/kg/h, SEM 29.184, 95% CI [-149.66, -24.474], *p* = 0.01, *n* = 15) as well as between V2 and V4 (mean difference − 72.667 µg/kg/h, SEM 22.988, 95% CI [-121.168, -24.165], *p* = 0.006, *n* = 18). Furthermore, paired t test of the mean LiMAx values of the patients who provided data on V1 and V2 also showed a significant improvement of the liver function after the two-week diet (mean difference − 27 µg/kg/h, SEM 9.05, 95% CI [-46.409, -7.591], *p* = 0.01, *n* = 15). The effect size for LiMAx improvement between V1 and V2 as well as V1 and V4 was large with a Cohen’s d of 0.8. For the LiMAx difference between V2 and V4, a Cohen’s d of 0.7 showed a medium effect size. However, Pearson’s correlation did not show significant associations between changes in weight and LiMAx values between V1 and V2 (*r* = -0.038, 95% CI [-0.540, 0.483], *p* = 0.892) as well as V1 and V4 (*r* = 0.140, 95% CI [-0.401, 0.609], *p* = 0.618).

In one patient (cllRYGB group), after an initial improvement of the liver function until three months post-surgery (V1: 403 µg/kg/h, V2: 420 µg/kg/h, V3: 431 µg/kg/h), a decline was observed at the last study visit (V4: 335 µg/kg/h) without concomitant clinical or laboratory anomalies. A fifth LiMAx test was performed one year postoperatively, which showed a moderate recovery to 387 µg/kg/h.

### Imaging-based assessments confirm a morphological improvement of hepatic steatosis after combined Diet and metabolic surgery

**Fig. 3:**
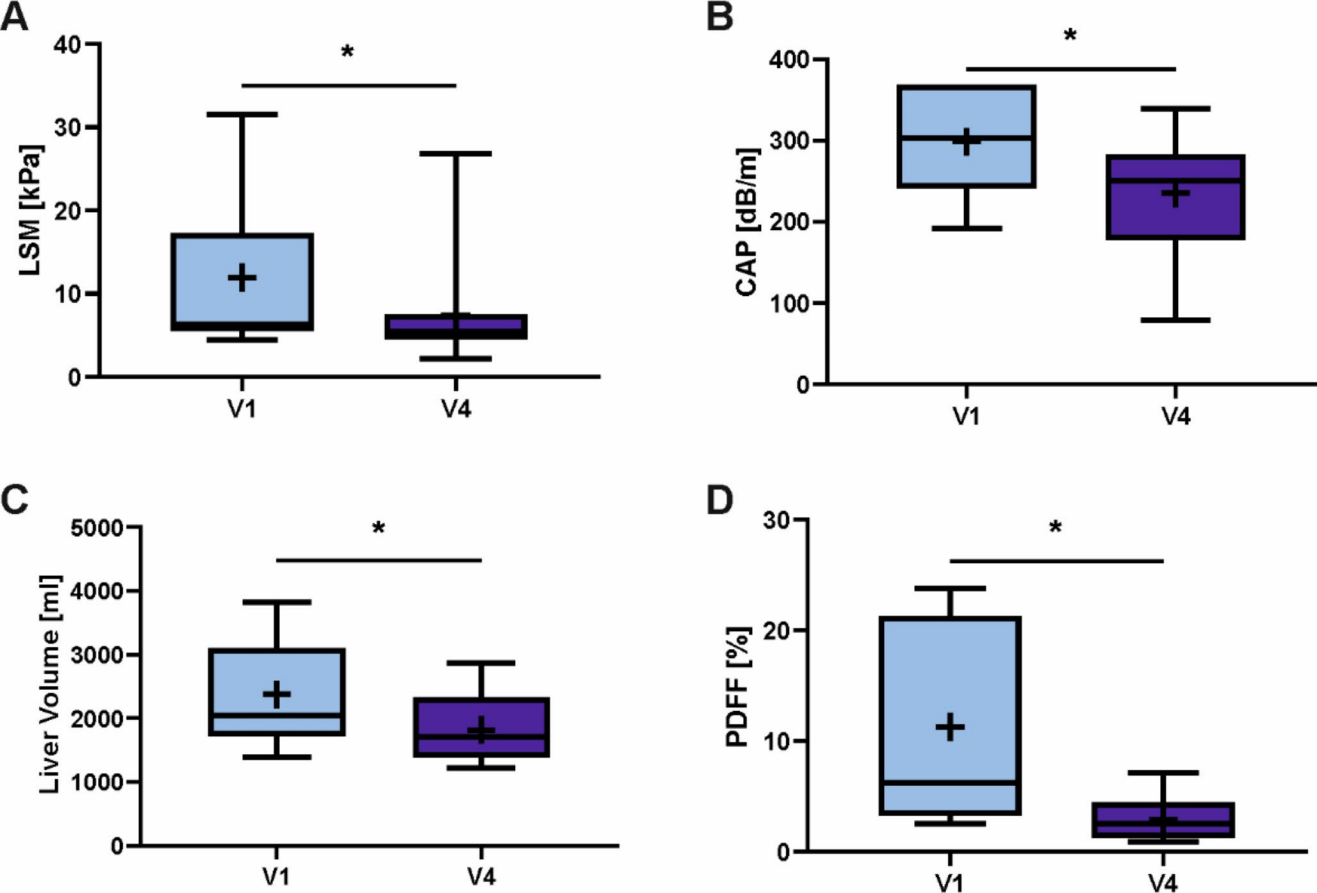
**Effects of Diet and Metabolic Surgery on Imaging-based MASLD Assessment.** Transient elastography and magnetic resonance imaging was performed at baseline and 6 months postoperatively after participants had undergone a two-week hypocaloric protein-rich diet and Roux-en-Y gastric bypass (RYGB) surgery to assess liver stiffness (**A**; *n* = 14; *p* = 0.032), hepatic steatosis (**B**; *n* = 14; *p* = 0.032), liver volume (**C**; *n* = 10; *p* = 0,005) and hepatic fat fraction (**D**; *n* = 12; *p* = 0,003). Data are expressed as boxplots with median as middle line and interquartile range (IQR) as ends of the box, minimum and maximum values as whiskers and a cross marking the mean value. Paired t test, *p* < 0.05 (*). Abbreviations: V1, baseline visit at participant enrolment in the study; V4, six months post-surgery; LSM, liver stiffness measurement; CAP, continuous attenuation parameter; PDFF, proton density fat fraction

MRI and TE examinations were performed at baseline and at the last study visit six months postoperatively to non-invasively assess the degree of MASLD. Data of patients who obtained measurements at both study visits were analyzed by two-sided paired t test (Fig. 3). Results from TE measurements could be obtained from 14 patients at V1 and V4. Both LSM (mean difference 4.55 kPa, SEM 1.896, 95% CI [0.453, 8.646], *p* = 0.032, *n* = 14) and CAP (mean difference 62.785 dB/m, SEM 26.158, 95% CI [6.272, 119.298], *p* = 0.032, *n* = 14) values were significantly improved at V4. MRI examinations were avalaible from 12 patients, but due to technical issues, liver volumetry data could only be obtained from 10 patients. Liver volume significantly decreased (mean difference 558.9 ml, SEM 151.955, 95% CI [215.153, 902.646], *p* = 0.005, *n* = 10) concomitantly with a significant reduction of the hepatic fat fraction measured by PDFF (mean difference 8.309%, SEM 2.205, 95% CI [3.453, 13.164], *p* = 0.003, *n* = 12). Effect sizes of TE measured improvements were medium (Cohen’s d of 0.6 for both LSM and CAP), but large for MRI-based measurements (Cohen’s d of 1.1 for liver volume and 1.0 for PDFF). Taken together, all imaging-based MASLD parameters improved 6 months after RYGB in line with the improved liver function measured by LiMAx test. The two surgical groups did not differ in any of the imaging-based measurements.

### Combined diet and metabolic surgery reduced the need for diabetes medication

At baseline, all participants were on medical treatment for T2DM (Table 1). Six months after RYGB, 67% (16 out of 24) patients did not require insulin and/or oral antidiabetic medication anymore.

## Discussion

This study provides evidence that the impaired liver function of patients with obesity and T2DM can be improved by a two-week hypocaloric protein-rich diet and furthermore by gastrointestinal bypass surgery.

Prevalence and severity of MASLD increase with BMI [21, 22]. This is even more pronounced through the presence of T2DM [22, 23]. In patients with a high degree of obesity, as in bariatric surgery candidates, liver biopsies revealed MASLD features in > 80% [24]. Besides increasing the overall cardiovascular risk [25], MASLD and the metabolic syndrome increase morbidity and mortality after major abdominal surgery [26, 27]. Since the majority of patients scheduled for metabolic/bariatric surgery are likely to have MASLD, it seems reasonable to preoperatively screen for impairments of the liver function and preferably apply means to reduce concomitant risks. At the beginning of our study, 50% of the participants had a pathological liver function capacity measured with the LiMAx test. This proportion is in line with findings of Alizai et al. who found pathologic LiMAx values in 61–64% of their bariatric surgery patients [14, 28]. Our group has reported the benefits of a two week hypocaloric, protein-rich diet prior to bariatric surgery [17]. In the current study, we sought to investigate the effects of such a diet on the liver function and could show significantly improved LiMAx values thereafter, implicating a restorative effect of the diet on the liver function in patients with obesity and T2DM. Thus, the implementation of similar dietary programs might also be beneficial in preparation for other major elective interventions or operations in this patient group. Further studies with a control group would be needed to validate our observations.

Six months after RYGB surgery, the liver function of our study participants increased even further. This was accompanied by significant decreases in the investigated imaging-based MASLD parameters. The observed liver function recovery is therefore likely to be explained by the reduced liver fat content [29], although our sample size was too small to identify a direct association. Improved LiMAx values after bariaric surgery were also reported by the group of Alizai and co-workers [28, 30]. However, in their first study they did not differentiate between patients receiving a RYGB or sleeve gastrectomy (SG) [30]. In another investigation the same group compared pre- and postoperative LiMAx tests after RYGB versus SG. The study methodology resembles very much that of our present investigations, also revealing an influence of preoperative weight on post-RYGB LiMAx recovery in a linear model analysis. However, RYGB patients in general did not significantly benefit from the operation in context of their liver function as opposed to SG patients [28]. In contrast to our study, the distribution of patients with type 2 diabetes in their study population was unbalanced, with significantly more type 2 diabetes patients in the SG group. But if T2DM was present in patients who underwent RYGB, this was associated with a decrease in LiMAx 6 months after surgery. In our study population, all patients had T2DM and the recovered liver function six months after surgery was also accompanied by a reduced need for pharmacological antidiabetes treatment. These findings are in agreement with a recent study that found an association between T2DM remission and the presence of low-grade hepatic steatosis in bariatric surgery patients [31] and further support the complex and dynamic interplay between MASLD and diabetes [32]. RYGB shows advantages over SG in terms of diabetes remission [33, 34]. By increasing the length of the BPL, even higher beneficial effects on T2DM could be expected [35]. In our study, no differences between the two surgical groups were observed. Yet, the group sizes in this substudy were not large enough to uncover group differences. The investigation of effects of different BPL lengths on T2DM will be the primary outcome of the superordinate MetaSurg study.

Since possible deteriorating effects of an increased BPL length on the liver function due to protein malnutrition have been discussed [36], it is important to remark that we did not see any adverse liver-related outcomes or other relevant complications in our study participants. Thus, both applied RYGB types are safe and effective with regard to the liver function.

### Limitations

This study did not contain a control group, meaning that changes in liver function cannot necessarily be attributed to the diet or the surgery, though it seems highly likely that these were indeed at the root of the changes. The small sample size and missing data (see Fig. 1, Study Flowchart) mainly due to entrance restrictions during the COVID-19 pandemic that prohibited routine check-ups and study visits constitute a further limitation. As a consequence, possible intergroup differences may remain hidden. Furthermore, monitoring of diet adherence relied on patients’ self-reports and a qualitative assessment by our nutritionists. However, this kind of preoperative diet-regimen is established practice in many bariatric/metabolic surgery centers nowadays and our study thus delivers real-life-data. The results of the patients’ weight history before and after diet indicated a good overall compliance.

## Conclusion

Patients with obesity and T2DM frequently suffer from an impaired liver function. However, metabolic surgery is a safe option in these patients to reduce body weight and liver fat and to improve T2DM management. In this patient group, a two-week hypocaloric protein-rich diet may be a beneficial means to improve liver function and to achieve at least some weight loss prior to abdominal surgery.

## Electronic supplementary material

Below is the link to the electronic supplementary material.

Supplementary Material 1

Supplementary Material 2

Supplementary Material 3

## Data Availability

The data that support the findings of the study are available from the corresponding author, Arne Dietrich, upon reasonable request.
