# Supplementary material for: A hypocaloric protein-rich diet before metabolic surgery improves liver function in patients with obesity and diabetes: A secondary analysis of a randomized clinical trial
Source: Langenbecks Arch Surg. 2025 Jan 13;410(1):36. doi: 10.1007/s00423-024-03600-9 (PMC11729132; doi:10.1007/s00423-024-03600-9)
Supplement: Supplementary file 2 — Supplementary Material 2 [file 423_2024_3600_MOESM2_ESM.pdf]

Online Resource 2 – Inclusion and exclusion criteria to the superordinate MetaSurg Study

Supplementary Information to:

A hypocaloric protein-rich diet before metabolic surgery improves liver function in patients with obesity and diabetes

A secondary analysis of a randomized clinical trial

Langenbeck's Archives of Surgery

Natalie Krönert*[1], Yusef Moulla*[1], ORCID: 0000-0002-5936-0217, Undine Gabriele Lange[1], ORCID: 0009-0009-5040-7128, Matthias Blüher[2,3], ORCID: 0000-0003-0208-2065, Nicolas Linder[4], Alexander Fuhrmann[4], Harald Busse[4], Anna Linder[4], Thomas Karlas[5], Johannes Wiegand[5], Roland Morgenroth[6], Lena Seidemann[1†], ORCID: 0000-0002-1531-7735, Arne Dietrich[1†]

[1]Department of Visceral, Transplant, Thoracic and Vascular Surgery, Leipzig University Hospital, Leipzig, Germany

[2]Department of Endocrinology, Nephrology, Rheumatology, Leipzig University Hospital, Leipzig, Germany

[3]Helmholtz Institute for Metabolic, Obesity and Vascular Research (HI-MAG) of the Helmholtz Zentrum München at the University of Leipzig and University Hospital Leipzig, Leipzig, Germany

[4]Department of Diagnostic and Interventional Radiology, Leipzig University Hospital, Leipzig, Germany

[5]Department of Oncology, Gastroenterology, Hepatology, Pneumology and Infectiology, Leipzig University Hospital, Leipzig, Germany

[6]Integrated Research and Treatment Center (IFB) Adiposity Diseases, University Hospital Leipzig, Leipzig, Germany

*Contributed equally as co-first authors

[†]Contributed equally as co-last authors

Correspondence: Prof. Dr. Arne Dietrich, Clinic for Visceral, Transplant, Thoracic and Vascular Surgery, Leipzig University Hospital, Liebigstr. 20, D-04103 Leipzig, Germany, arne.dietrich@medizin.uni-leipzig.de

Inclusion Criteria

- Body mass index (BMI) ≥27 to ≤60 kg/m2
- Type 2 diabetes mellitus (T2DM)
  - for patients BMI ≥35 kg/m2: any T2DM
  - for patients BMI < 35 kg/m2:
    - unsatisfactory medical non-insulin treatment (multiple medications, HbA1c >6.5%, hypo- or hyperglycaemias) or
    - planned insulin treatment or ongoing insulin treatment
- males and females, age ≥18 years
- written informed consent

Exclusion Criteria

- any chronic inflammatory or malignant disease
- type 1 diabetes (C-Peptid < 0,5 µg/l, GAD-Ab or ICA-Ab positive)
- peptic ulcer
- contraindication for general anaesthesia
- drug or alcohol abuse
- untreated thyroid dysfunction
- pregnancy or breast feeding woman
- fertile women (within two years of their last menstruation) without appropriate contraceptive measures (implanon, injections, oral contraceptives, intrauterine devices, partner with vasectomy) while participating in the investigation
- expected non-compliance
- participation in other interventional trial
