# Supplementary material for: A hypocaloric protein-rich diet before metabolic surgery improves liver function in patients with obesity and diabetes: A secondary analysis of a randomized clinical trial
Source: Langenbecks Arch Surg. 2025 Jan 13;410(1):36. doi: 10.1007/s00423-024-03600-9 (PMC11729132; doi:10.1007/s00423-024-03600-9)
Supplement: Supplementary file 3 — Supplementary Material 3 [file 423_2024_3600_MOESM3_ESM.pdf]

Online Resource 3 – Diet instructions for study participants

Supplementary Information to:

A hypocaloric protein-rich diet before metabolic surgery improves liver function in patients with obesity and diabetes

A secondary analysis of a randomized clinical trial

Langenbeck's Archives of Surgery

Natalie Krönert*[1], Yusef Moulla*[1], ORCID: 0000-0002-5936-0217, Undine Gabriele Lange[1], ORCID: 0009-0009-5040-7128, Matthias Blüher[2,3], ORCID: 0000-0003-0208-2065, Nicolas Linder[4], Alexander Fuhrmann[4], Harald Busse[4], Anna Linder[4], Thomas Karlas[5], Johannes Wiegand[5], Roland Morgenroth[6], Lena Seidemann[1†], ORCID: 0000-0002-1531-7735, Arne Dietrich[1†]

[1]Department of Visceral, Transplant, Thoracic and Vascular Surgery, Leipzig University Hospital, Leipzig, Germany

[2]Department of Endocrinology, Nephrology, Rheumatology, Leipzig University Hospital, Leipzig, Germany

[3]Helmholtz Institute for Metabolic, Obesity and Vascular Research (HI-MAG) of the Helmholtz Zentrum München at the University of Leipzig and University Hospital Leipzig, Leipzig, Germany

[4]Department of Diagnostic and Interventional Radiology, Leipzig University Hospital, Leipzig, Germany

[5]Department of Oncology, Gastroenterology, Hepatology, Pneumology and Infectiology, Leipzig University Hospital, Leipzig, Germany

[6]Integrated Research and Treatment Center (IFB) Adiposity Diseases, University Hospital Leipzig, Leipzig, Germany

*Contributed equally as co-first authors

[†]Contributed equally as co-last authors

Correspondence: Prof. Dr. Arne Dietrich, Clinic for Visceral, Transplant, Thoracic and Vascular Surgery, Leipzig University Hospital, Liebigstr. 20, D-04103 Leipzig, Germany, arne.dietrich@medizin.uni-leipzig.de

Dear patient,

You are about to undergo surgery that will reduce your weight in the long term and significantly improve your health prognosis. By following a 14-day protein diet, you will help reduce perioperative risks.

The main goal of this preparatory dietary change is to shrink the existing fatty liver to provide for better surgical conditions. A strictly low-calorie, low-fat, low-carbohydrate diet will reduce the metabolic burden of the liver. In addition, the predominantly liquid diet offers you the opportunity to get used to small portion sizes already prior to the operation. You will need more time per meal and you will avoid unnecessary swallowing of air. This is considered a useful training.

Patients with diabetes mellitus and a drug therapy should consult the treating diabetologist or dietitian before starting the preparatory diet because of a possibly necessary adjustment of the medication.

To ensure sufficient protein intake and prevent unnecessary muscle loss, we recommend that you take one of the protein preparations listed (preferably as a shake and thus as a meal replacement), which you can purchase at pharmacies or on the internet under the respective name. All of the products mentioned are of the same high quality, strictly low in lactose, cholesterol and sodium and gluten-free. They are neutral in taste and can be stirred into almost all hot and cold liquids. The consistency of the food and beverages is hardly changed by this; there should be no lump formation.

- 2 weeks before the date of your operation, start the protein diet by replacing all 3 main meals and 1 snack with such a protein drink every day. The instructions should be implemented as follows:

- 2-3 tablespoons of protein powder in 200- 250 ml of low-fat milk/ buttermilk/ water.

- Addition of small amounts of natural flavors (e.g. cinnamon, lemon juice, vanilla, mint, 1- max. 2 tsp. soluble cocoa, coffee or matcha tea powder) is possible.

You will need a total of approx. 1 kg of one of these products for the 14-day protein phase

- Fresubin Protein POWDER (Fresenius Kabi Deutschland GmbH)

- Nestlé Protein Instant (Nestlé Health Science)

- MetaX AdPro 104 (MetaX GmbH Friedberg)

- Bariatric Advantage- Whey (Metagenics Deutschland)

- Fitforme- Pure Whey Eiweiß (Fit for me B.V.)

Dosage: 10- 12 tablespoons / day

The following recommendations apply for the 14 preoperative days (i.e. before surgery):

- daily intake of the protein concentrate (10-12 tablespoons) as a meal replacement

- plan a total of 4 meals a day (in the form of the protein drinks)

- vegetables can be eaten in unlimited quantities throughout the day - as raw vegetables, also with homemade low-fat herb curd cheese or yogurt dressing for dipping, mixed salads with homemade dressing made of low-fat yogurt or buttermilk (do not use oil dressing!)

- process vegetables into vegetarian stews, soups, or a flavorful vegetable stir-fry (grilled, braised, or steamed); be sure to prepare them fat-free

- use fresh herbs and spices for flavorful meals

- low-fat natural dairy products with max. 1.5% fat such as milk, buttermilk, natural yoghurt and low-fat curd cheese can also be used

- low-fat natural milk alternatives with max. 1.5% fat such as soy, nut, almond or coconut drink (please do not use oat or rice milk) are possible

- refine yogurt and quark with mint, lemon, cinnamon, natural vanilla flavoring or soluble cocoa or coffee powder, for a sweetish-tart taste you can use matcha tea powder

- make sure that you drink enough (at least 2 liters a day), unsweetened fruit and herbal tea, water, pure vegetable juices and 1-2 cups of bean coffee or malt coffee are possible

- 5 days before the surgery, use a little more milk, vegetable broths, water or buttermilk to prepare your food to achieve a more liquid consistency to be able to absorb it through a drinking straw

- at the same time (i.e. 5 days before surgery), also start taking your daily multivitamin (in the form of a capsule, chewable tablet or powder)

Avoid:

- processed dairy products (e.g. fruit yogurts/yogurt preparations) with added fruit or cream, or full-fat levels

- fish, poultry and other meats, sausages and cheese

- all foods high in carbohydrates such as potatoes, cereal products (bread and bakery products, pasta, rice...), sweets and sweeteners (sugar, honey, agave syrup, syrup), all types of fruit (including juices, dried fruit), spreads (jam, honey, chocolate cream)

- all fatty foods such as nuts, all oils, butter, margarine, lard as well as avocado, olives

- all highly processed foods and convenience foods, such as instant soups, canned foods, and other instant preparations

- reduce the use of salt to a minimum
